# Imaging in Acute Coronary Syndrome

**DOI:** 10.4061/2011/461720

**Published:** 2011-12-26

**Authors:** Alexander Hirsch, Robin Nijveldt, Igor Klem

**Affiliations:** ^1^Department of Cardiology, Academic Medical Center, University of Amsterdam, B2-238, Meibergdreef 9, P.O. Box 22660, 1100 DD Amsterdam, The Netherlands; ^2^Department of Cardiology, VU University Medical Center, De Boelelaan 1117, P.O. Box 7057, 1007 MB Amsterdam, The Netherlands; ^3^Duke Cardiovascular Magnetic Resonance Imaging Center, Duke University Medical Center, Box 3934 2100 Erwin Road, Durham, NC 27710, USA

Acute coronary syndrome is a leading cause for hospital admissions both in Europe and the United States resulting in high medical care expenses. During the past 25 years, there have been important advances in the therapeutic options for patients with an acute coronary syndrome with improved medical therapy and the introduction of percutaneous coronary intervention. However, despite the reduction of morbidity and mortality due to various refinements in medical treatment and technology, there are still opportunities to improve outcome in the first months after the event and on the longer run. Accurate recognition of patients that can benefit from additional therapies is, therefore, crucial in the therapeutic management of patients after an acute coronary syndrome. 

The standard of care of patients with acute coronary syndrome includes a stepwise approach including different imaging modalities to make a final diagnosis, with rest echocardiography as the most common of the techniques. Recently, new imaging techniques have emerged resulting in a wide array of choices available to cardiologists to improve clinical decision making. These new modalities can be helpful in several aspects including the detection and differential diagnosis of acute coronary syndromes, guiding clinical decision making and improving risk stratification after an event. This is an exciting and a rapidly expanding field of investigation. 

In this special issue, several interesting articles in relation to this topic are published for the readership of Cardiology Research and Practice. The first paper is an excellent review by L. Budge and P. Salerno on the role of the cardiovascular magnetic resonance in the evaluation of patients presenting with suspected or confirmed acute coronary syndrome. The authors clearly discuss the role of cardiovascular magnetic resonance in the emergency department for the timely and accurate identification of non-ST-elevation acute coronary syndrome patients. They also highlight the application of cardiovascular magnetic resonance in patients after ST-segment-elevation myocardial infarction because of the identification of complications postmyocardial infarction and the important prognostic information this imaging technique can provide. The second paper is a review by T. Kubo et al. that concerns the current status of optical coherence tomography, a more recently developed intracoronary imaging technique. In comparison with intravascular ultrasound, optical coherence tomography has a high resolution and offers microscopic visualization of coronary plaques. The authors clearly review the current literature and discuss the implications for clinical practice. The following paper is a clinical study by the same research group in which lesion characteristics detected by intravascular ultrasound were compared with images obtained with optical coherence tomography in 104 patients with unstable angina. This paper provides useful data because intracoronary imaging techniques are more and more used in daily practice. 

The following three papers are all case reports. The first report is about two patients who both presented with stent fracture after everolimus-eluting stent implantation. Stent fracture has emerged as a potential mechanism of drug-eluting stent failure that is associated with in-stent restenosis. In the second case report, a patient is presented with an anomalous left main coronary artery originating from right aortic sinus having retroaortic course that was complicated by a significant atherosclerotic narrowing. This patient was admitted with an acute coronary syndrome that was treated by percutaneous coronary intervention. In the last paper a case of acute thrombosis of two simultaneous coronary arteries in a young adult is described. The patient showed elevated homocysteine levels associated with homozygosity for the C677T gene mutation of methylenetetrahydrofolate reductase, a genetic condition that is associated with early coronary artery disease onset.

We hope that the readers will find this special issue of interest.

*Alexander Hirsch* *Robin Nijveldt* *Igor Klem*

